# Ineffective Erythropoiesis in ****β****-Thalassemia

**DOI:** 10.1155/2013/394295

**Published:** 2013-03-28

**Authors:** Jean-Antoine Ribeil, Jean-Benoit Arlet, Michael Dussiot, Ivan Cruz Moura, Geneviève Courtois, Olivier Hermine

**Affiliations:** ^1^Centre National de la Recherche Scientifique-Unité Mixte de Recherche 8147, Université Paris V, René Descartes, Hôpital Necker, Paris, France; ^2^Département de Biothérapie, Faculté de Médecine Paris Descartes, Sorbonne Paris-Cité et Assistance Publique—Hôpitaux de Paris, Hôpital Necker, Paris, France; ^3^Fondation Imagine, Institut des Maladies Génétiques, Faculté de Médecine Paris Descartes, Sorbonne Paris-Cité et Assistance Publique—Hôpitaux de Paris, Hôpital Necker, Paris, France; ^4^Laboratoire d'Excellence des Globules Rouges (GR-ex), Paris, France; ^5^Fondation Imagine, Université Paris Descartes-Sorbonne Paris Cité, Paris, France; ^6^Service de Médecine Interne, Faculté de Médecine Paris Descartes, Sorbonne Paris-Cité et Assistance Publique—Hôpitaux de Paris, Hôpital Européen Georges Pompidou, Paris, France; ^7^INSERM U 699, Hôpital Bichat, Université Paris Diderot, Paris, France; ^8^Service d'Hématologie, Faculté de Médecine Paris Descartes, Sorbonne Paris-Cité et Assistance Publique—Hôpitaux de Paris Hôpital Necker, Paris, France

## Abstract

In humans, **β**-thalassemia dyserythropoiesis is characterized by expansion of early erythroid precursors and erythroid progenitors and then ineffective erythropoiesis. This ineffective erythropoiesis is defined as a suboptimal production of mature erythrocytes originating from a proliferating pool of immature erythroblasts. It is characterized by (1) accelerated erythroid differentiation, (2) maturation blockade at the polychromatophilic stage, and (3) death of erythroid precursors. Despite extensive knowledge of molecular defects causing **β**-thalassemia, less is known about the mechanisms responsible for ineffective erythropoiesis. In this paper, we will focus on the underlying mechanisms leading to premature death of thalassemic erythroid precursors in the bone marrow.

## 1. Introduction 

Normal human adult hemoglobin (Hb) A (HbA) consists of two pairs of globin chains,*α*_2_ *β*_2_, of which synthesis is normally tightly coordinated to ensure equal production. *β*-thalassemia, one of the most common inherited hemoglobinopathy in the world, is due to autosomal mutations in the gene encoding *β*-globin which induce an absence or low-level synthesis of this protein in erythropoietic cells [1]. The consequence of these mutations is an imbalance of *α*/*β*-globin chain synthesis, mostly evident in the homozygous forms, leading to the accumulation of free *α*-globin chains forming highly toxic aggregates [2]. Thalassemic patients suffer from anemia resulting from shortened red blood cell (RBC) survival, by hemolysis, and erythroid precursors premature death in bone marrow (ineffective erythropoiesis).

The first description of thalassemia was reported by Dr. Thomas Cooley in 1925. There are a multiplicity of different genetic mutations in *β*-thalassemia that give rise to a clinically heterogeneous spectrum ranging from asymptomatic expression (thalassemia minor) and mild clinical anemia (thalassemia intermedia) to classical, fatal Cooley's anemia. The term “Cooley's anemia,” now termed *β* ^0^-thalassemia major (TM), has been used synonymously with clinically severe forms of *β*-thalassemia, characterized by a very high *α*/non-*α* chains ratio, severe ineffective erythropoiesis, and dependence on RBC transfusions to sustain live. Regular transfusions (average every month) expose these patients to iron overload and its live threatening systemic consequences, which require iron chelation [1].

It is well established that the *α*/non-*α* ratio correlates with the severity of disease [1, 3]. However, genotypic variability impairing globin chain synthesis at known loci is often insufficient to explain the heterogeneity in clinical phenotypes of individual patients with the same genotype, suggesting that other genetic modulations might exist [1, 4]. The molecular mechanisms underlying the heterogeneity and occasional severity of the syndrome remain obscure and are not the object of this paper.

The pathophysiology of *β*-thalassemia has been the subject of several extensive reviews, particularly on its molecular and genetics basis [5]. In this paper, we will focus on the mechanisms leading to end-stage maturation blockade and thalassemic erythroid precursors premature destruction in the bone marrow. Their understanding will probably be the key for developing novel therapeutic approaches improving anemia in *β*-thalassemia. In order to describe mechanisms underlying ineffective erythropoiesis, we will first summarize the current knowledge on normal hemoglobin synthesis and normal erythropoiesis. 

## 2. Hemoglobin Synthesis

Two distinct globin chains *α* and *β* (each carrying an individual heme molecule) interact to form hemoglobin dimers *α*/*β*, and two dimers combine to form a hemoglobin tetramer *α*_2_ *β*_2_: the functional form of hemoglobin carrying oxygen.

Excepted the very first weeks of embryogenesis, in which zeta chains are produced, one of the globin chains is *α* and the second chain is called “non-*α*.” The main (98%) hemoglobin type in the normal human adult consists of two *α* and two *β* chains (*α*_2_ *β*_2_ HbA); the minor type (2%) consists of 2*α* and 2*δ* chains (*α*_2_ *δ*_2_ HbA2). Usually, globin chains synthesis is relatively balanced, even if some studies report a slight excess of *α* chains as a soluble pool [3, 6, 7]. In contrast, fetal erythrocytes contain another type of hemoglobin consisting of 2*α* and 2*γ* chains (*α*_2_ *γ*_2_ HbF), which can attract more oxygen effectively from the maternal blood. 

Globin chains originating from a common ancestral type display a varying degree of homology. Two clusters of globins genes are known: the first one on chromosome 16 for *α*-like genes (two *α*-globin genes, *α*_1_ and *α*_2_, and two zeta genes) and the second one on chromosome 11, for *β*-like genes. The 5′ to 3′ order of *β*-like globin genes on chromosome 11 (*ε*-^G^ *γ*-^A^ *γ*-*δ*-*β*, two *γ* genes) reflects their sequential activation and silencing, during the transition from embryonic to fetal and from fetal to adult “hemoglobinopoiesis,” called “hemoglobin ontogeny” or “hemoglobin chain switch.”

## 3. Erythropoiesis

Erythropoiesis is defined as the pathway producing mature RBC from hematopoietic stem cells. This process includes several steps restricting differentiation and proliferation of cells which undergo this erythroid program, depending on sequential and specific erythroid gene expression. Erythropoiesis is regulated by combined effects of microenvironment and growth factors that promote survival, proliferation, and/or differentiation of erythroid progenitors and nuclear factors that regulate transcription of genes involved in survival and establishment of the erythroid phenotype. RBC production is orchestrated by a complex network of transcription factors, among which GATA-1, the master gene of erythropoiesis, positively regulates specific erythroid genes such as erythropoietin receptor (EpoR), glycophorin (GpA), and globin chains. Moreover, together with the transcription factor STAT5 (activated through EpoR activation by erythropoietin (Epo)), GATA-1 induces the expression of the anti-apoptotic protein Bcl-xL [8]. 

Committed erythroid progenitors differentiate into the first morphologically identified cell of the erythrocyte lineage: the proerythroblast. Next steps of erythroid differentiation are accompanied by temporally regulated changes in cell surface protein expression, reduction in cell size, progressive hemoglobinization, and nuclear condensation (successively called basophilic erythroblast, polychromatophilic erythroblast, and acidophilic erythroblast, the last nucleated cell of the mammalian erythrocyte lineage), which culminate in reticulocytes cells by nucleus, RNA, and mitochondria extrusion. In addition, erythroid maturation requires a transient activation of caspase-3 at the basophilic stage and translocation into the nucleus of the inducible heat shock protein 70 (Hsp70) to protect GATA-1 from caspase-3 cleavage [9, 10].

This process occurs within the erythroblastic island, in which a macrophage is surrounded by erythroblasts at all stages of maturation [11, 12]. The production of erythrocytes is the largest quantitative output of the hematopoietic system with estimated production rates of 2 × 10^11^ erythrocytes per day. The program of erythroid proliferation and differentiation must be positively and negatively regulated to ensure a continuous but tightly controlled production of RBC.

### 3.1. Positive Regulation of Erythropoiesis

Erythropoiesis is controlled by the combined effect of two major cytokines, stem cell factor (SCF) and Epo. SCF induces proliferation and survival and slows down differentiation of early erythroid progenitors and precursors towards the basophilic erythroblast stage. Epo is responsible of the finely tuned homeostatic control of erythrocyte numbers by tissue oxygenation. Interaction of Epo with the EpoR induces, through JAK2 activation, multiple signalling pathways involving PI3 kinase, Akt, and STAT5, which prevent apoptosis, supporting erythroid progenitors proliferation and allowing erythroid program to occur [13–15]. 

### 3.2. Negative Regulation of Erythropoiesis by Apoptosis

The negative regulation of erythropoiesis is mainly due to apoptosis, a fundamental cellular mechanism allowing clearance of unneeded or potentially dangerous cells. Apoptotic programs require the action of a family of cysteine-dependent and aspartate-specific proteases called caspases. Two classes of caspases are described: initiators (caspase-8 and -9) and effectors (caspase-3 and -7) [16, 17]. Caspase-8 is activated by the death receptor pathway after cell surface receptor-ligand interaction [18]. In contrast, caspase-9 is activated by events causing intracellular damages and alterations in mitochondrial membrane potential (i.e., the mitochondrial pathway) [19, 20]. Activated caspase-8 and caspase-9 then activate effectors such as caspase-3 that cleaves GATA-1, Tal-1 [21, 22], and proteins involved in cytoplasm, nucleus, and DNA integrity, which allow the cell death program to occur. 

### 3.3. Role of Cell Death Receptors and Epo

Death receptors of the TNF receptor (TNF-R) superfamilies (Fas-L, TNF-*α*, TRAIL) activate the extrinsic apoptotic pathway. Fas and Fas-L are expressed in cultured erythroblasts, but controversies regarding the level and differentiation stage at which they are expressed have been reported. Some studies suggest the existence of a negative regulatory feedback operating at low Epo level in a paracrine pathway. In this system, Fas-L expressing mature erythroblasts displays cytotoxicity against immature erythroblasts expressing Fas [23, 24]. Epo is able to partially protect immature erythroid cells from Fas-mediated apoptosis. Fas and Fas-L are therefore major regulators of erythropoiesis. In addition Fas/Fas-L interaction results, through caspase-8 activation, in GATA-1 cleavage which blocks erythroid differentiation and maturation [25].

The control of mature RBC production may be summarized as follow: at low doses of Epo, cells die by apoptosis; at intermediary doses, cells are arrested in their differentiation and maturation (through GATA-1 cleavage) or enter a program of apoptosis depending on the number of mature erythroblasts in the bone marrow; at high dose, erythroid progenitors and precursors pursue their maturation independently of the number of mature erythroid precursors. 

### 3.4. Role of Caspases and Hsp70 in Differentiation and Maturation of Erythroid Cells

The terminal differentiation of erythroid cells exhibits some similarities with apoptosis, such as reduction in cell size, chromatin condensation, and degradation of nuclear components. A transient activation of caspases by the mitochondrial pathway has been shown, by our group and others, to be required for erythroid cells differentiation but to not induce neither GATA-1 cleavage nor apoptosis. We have more recently reported that Hsp70, an ubiquitous chaperone constitutively expressed during erythroid differentiation, protects GATA-1 in the nucleus from caspase-3-mediated proteolysis during caspase activation. These results strongly indicate that Hsp70 is another key erythroid antiapoptotic protein protecting GATA-1 from caspase-3-mediated cleavage and consequently allowing Bcl-x_L_ expression [9, 10]. 

## 4. *β*-Thalassemia Ineffective Erythropoiesis

### 4.1. Evidences for an Ineffective Erythropoiesis in *β*-Thalassemia

Dyserythropoiesis in *β*-thalassemic patients was suspected for a long time since it is largely recognized that many patients with an inadequate transfusional regimen have a dramatic expansion of the hematopoietic marrow and extramedullary hematopoiesis, which can lead to extensive bone deformity and/or bone marrow mass and splenomegaly. Erythrokinetic assays, done in the 50′s, showed that the rate of peripheral RBC destruction in *β*-thalassemia was insufficient to explain severe anemia [26, 27]. Then, ferrokinetic studies done in the 70′s, studying incorporation of ^59^Fe into newly formed RBC, suggested that probably 60%–80% of erythroid progenitors were arrested in proliferation and/or underwent death [28]. 

The bone marrow of patients suffering from *β*-thalassemia contains five to six times the number of erythroid precursors observed in healthy controls [29], with increased basophilic and polychromatophilic erythroblasts and decreased orthochromatic erythroblasts [29–32]. Moreover, it has been shown that *β*-thalassemic bone marrow erythroblasts contain electron-dense alpha-globin inclusion (aggregates) beginning at early polychromatophilic stages, which increase in size and frequency during subsequent maturation [33].

**Figure 1:**
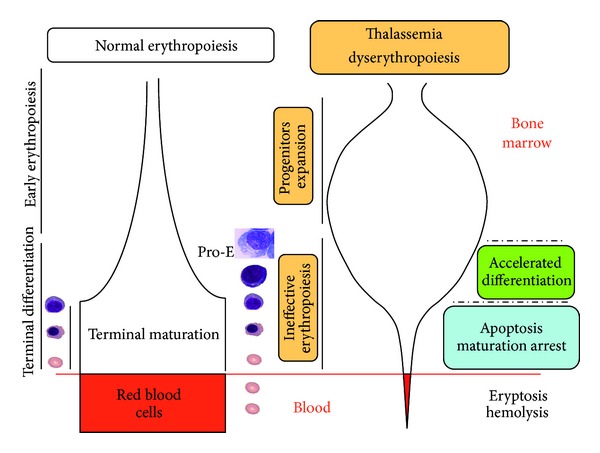
Difference between normal and *β*-thalassemia ineffective erythropoiesis. Erythropoiesis is the pathway producing mature RBCs from hematopoietic stem cells, including several proliferation and differentiation steps. Erythroid differentiation is accompanied by temporally regulated changes in cell surface protein expression, reduction in cell size, progressive hemoglobinization, and nuclear condensation and extrusion. *β*-thalassemia dyserythropoiesis in human is characterized by expansion of very early erythroid precursors (proerythroblasts and earlier stages) and then ineffective erythropoiesis. Ineffective erythropoiesis defines the suboptimal production of mature erythrocytes from a proliferating pool of immature erythroblasts characterized by (1) accelerated erythroid differentiation, (2) maturation blockade at the polychromatophilic stage, and (3) death of erythroid precursors.

Taken together, these results resume the findings of *β*-thalassemia dyserythropoiesis in human: expansion of very early erythroid precursors (proerythroblasts and earlier stages) and then ineffective erythropoiesis. Ineffective erythropoiesis defines the suboptimal production of mature erythrocytes from a proliferating pool of immature erythroblasts. It is thus characterized by (1) accelerated erythroid differentiation, (2) maturation blockade at the polychromatophilic stage, and (3) death of erythroid precursors [29, 30, 32, 34, 35] (Figure 1).

Although early erythroid progenitors expansion is believed to be due to a dramatic increased in Epo level as a result of the anemic state feedback [36], other mechanisms, yet not known, might be involved. In addition, precise pathophysiological mechanisms of accelerated erythroid differentiation and maturation arrest are still unknown. However, mechanisms underlying cell death were more studied and we intend at that point to review the different players of this death “game.”

### 4.2. Enhanced Apoptosis Is a Key Feature of Ineffective Erythropoiesis in Human *β*-Thalassemia

It was evidenced in the 90s that *β*-TM erythroid precursors, but neither lymphoid and nor myeloid precursors underwent increased apoptosis (among 3- to 4-fold increased compared to healthy controls) as detected in human and mice bone marrow by an increase in DNA laddering (a sign of enhanced nucleosomal DNA cleavage, occurring specifically during apoptosis) and then confirmed by TUNEL labelling and exposure of phosphatidyl serine assessed by Annexin V labelling by FACS analysis [29, 30, 32]. *In vitro* findings corroborate the reduced cell expansion in *β*-TM erythroid cultures and enhanced apoptosis at the polychromatophilic stage of differentiation [32]. 

In spite of the markedly increased rate of apoptosis of *β*-thalassemic erythroid precursors, BM smears of these patients do not show high increased number of dying erythroblasts. Indeed, 15% to 20% of bone marrow erythroid precursors (CD45^−^/CD71^+^) present apoptotic features in aspirates [29, 30, 32]. This paradox might be explained by increased phagocytosis of abnormal precursors erythroblasts expressing phosphatidyl serine by bone marrow macrophages whose number and activation are enhanced, respectively, by about 2-fold in TM [29, 37, 38]. As a consequence, the delivery of thalassemic RBCs to the peripheral blood in the *β*-TM major patients is much reduced. 

### 4.3. Apoptotic Pathways Involved in *β*-Thalassemia Ineffective Erythropoiesis

Studies of apoptotic death receptor pathways have shown that Fas and FasL are coexpressed early and at all stages of terminal differentiation. Both proteins are downregulated in bone marrow or spleen in proerythroblast and basophilic cells in *β*-thalassemic mice compared to control mice *in vivo*. No statistically difference was found in more mature cells. This down regulation in Fas/FasL expression might be a marker of erythropoietic stress [39] and might explain at least in part erythroid expansion. 

Regarding the intrinsic apoptotic pathway, it was expected that it would have been also involved because it could be induced by cellular oxidant injury. Nevertheless, the involvement of this mitochondrial pathway has not been evidenced to date in *β*-thalassemia [40]. 

### 4.4. Role of Oxidant Injury in *β*-Thalassemia Ineffective Erythropoiesis

#### 4.4.1. Excess of Unmatched Globin Chains Generates Reactive Oxygen Species (ROS)

Occurrence of increased death at the polychromatophil stage of differentiation in TM [32] coincides with the stage of intense hemoglobinization [4, 27, 41, 42].

It could partially be explained by accumulation and precipitation of the unmatched *α*-globin chains at this stage, forming aggregates [33]. Indeed, there is several evidence that membrane components oxidation might play an important role in *β*-thalassemia pathophysiology [5, 43]. Free *α*-globin which is highly unstable and bound to heme and iron could generate reactive oxygen species (ROS) that damage cellular proteins, lipids, and nucleic acids [44, 45]. Data describing ROS production during erythroid differentiation in thalassemia are scarce [46]. A significant increase in ROS production both in early and late erythroid precursors compared to normal erythroblasts was evidenced in *β*-thalassemia intermedia [35] or *β*-thalassemia/HbE [34]. Thus it was speculated that the excess of ROS, by damaging components of RBC, might reduce lifespan of these cells and cause a premature RBC clearance by hemolysis (a passive process) [45]. However there is no robust evidence that ROS production is the direct cause of increased apoptosis in *β*-thalassemic erythroid cell precursors [5]. The observation that there are no differences [35] or a dramatic decrease [34] in ROS levels during thalassemic erythroid differentiation pleads against a direct mechanism leading to high ROS levels and apoptosis, since significant increased apoptosis was only observed in late differentiation stage (polychromatophilic stage and after) [32]. Actually, the direct link between increase of ROS and apoptosis has never been demonstrated in normal human erythroid cells. However in other cell models, ROS activate apoptosis signal regulating kinase 1 (ASK1) and Jun-kinase which can induce apoptosis (extrinsic or intrinsic signaling pathway) [47]. ROS are also known to trigger the intrinsic apoptotic cascade via interactions with proteins of the mitochondrial permeability transition complex [48]. 

In conclusion, even if increased ROS levels might be an actor of erythroid cell precursors death by inducing damages of erythroid cell components, the pathophysiological relationship between apoptosis and accumulation of the unmatched *α*-globin chain in erythroblasts needs to be clarified. To date, no data provide clear evidences that the apoptotic mechanism involves ROS. On the other hand, the role of ROS in accelerated cell differentiation, another characteristic of ineffective erythropoiesis, is questionable [34] since antioxidants inhibit the *in vitro* erythroid progenitors differentiation from mice fetal liver [49]. 

#### 4.4.2. Damaging Membrane Structures: A Cause of Ineffective Erythropoiesis?

Unmatched *α*-globin chains and ROS production could induce alterations in membrane deformability, stability, and cellular hydration in addition to damages to cytoskeleton, explaining peripheral hemolysis [50]. Protein 4.1, a major component of the skeleton controlling its crosslinking, is partially oxidized in *β*-thalassemia's mature erythroblasts [51]. *α*-globins accumulation can occur even from the proerythroblast stage and such deposits were shown to colocalize with areas of defective assembly of the membrane skeletal proteins spectrin and protein 4.1 [31, 52]. Furthermore, very early in erythropoiesis, a defective assembly of the transmembrane band 3 protein was reported, which was not spatially related to *α*-globin deposits. This band 3 defect seems to disappear at the intermediate or late normoblast stage, suggesting either that the defect was temporary or that it severely affected band-3-deficient erythroid precursors which died and were removed. Furthermore, oxidant injury led to clustering of band 3, which in turn produced a neoantigen that bound IgG and complement [5, 53]. Extra-membranous IgG/complement complex provided signals for macrophages to remove such affected erythroid precursors and RBC. 

All these studies showed that *β*-thalassemic RBC membranes exhibited abnormalities in membrane skeletal proteins. Thus, it could also be postulated that the accumulation of *α* chains destabilizes the membrane and could participate in ineffective erythropoiesis, but it cannot be the unique explanation of the major increase of apoptosis [30, 31].

#### 4.4.3. The *α*-Hb-Stabilizing Protein (*AHSP*) Is an Erythroid-Specific Private Chaperone Protein That Specifically Binds *α*-Hb

Recently, an **α**-hemoglobin-stabilizing protein (*AHSP*) was identified. This protein specifically binds to and stabilizes free **α**chains. *AHSP* is a 102 amino acid erythroid-specific protein induced by the essential erythroid transcription factor GATA-1 [54]. *AHSP* is abundant in late-stage erythroid precursors, in which its expression kinetics match with *α*-globin ones [55]. Stabilization of native folded *α*-globin by *AHSP* might be particularly important when heme quantity is limited, for example, during iron deficiency. Indeed, the presence of functional iron response element (IRE) in the 3′ untranslated region of human *AHSP* mRNA stabilizes this transcript in low iron conditions [56, 57].

A second *AHSP* function is to detoxify the excess of *α*-globin chains. Protein interaction screening has shown that *AHSP* binds free *α* globin and *α*-hemoglobin (unmatched **α**-globin chains bound to heme) (*α*-Hb). Subsequent investigations provide evidence that *AHSP* acts as a protein-specific molecular chaperone to fold and stabilize *α*-globin for HbA synthesis [58] and to protect erythroid cells against the deleterious effects of excess free *α*-Hb [45]. *AHSP* stabilizes the structure of *α*-Hb and detoxifies it by inhibiting the ability of heme iron to participate in chemical reactions that generate damaging ROS in RBC [45, 54, 59, 60]. In *β*-thalassemia mice model, loss of *AHSP* deficiency exacerbates anemia [45]. *AHSP* ^−/−^ mice present mild hemolytic anemia and hemoglobin precipitation in RBCs [45]. *AHSP* ^−/−^ mice exhibited also an elevated proportion of immature erythroid precursors, a maturation arrest, and excess of apoptosis [45]. This effect could also reflect ineffective erythropoiesis as a consequence of excess of free *α* chains. 

The role of *AHSP* in human disease remains an open question. Naturally occurring mutations that ablate *AHSP* expression or alter the protein structure are rare [61, 62]. However, in human studies it was found that quantitative variation in *AHSP* expression between different individuals is extremely common [61]. Causal relationships between decreased *AHSP* expression and severity of thalassemic syndromes have not been established unequivocally [57]. 

### 4.5. Role of Heme and Heme Inhibitors in *β*-Thalassemia Ineffective Erythropoiesis

Excess of *α*-globin chains are associated with reduced heme production in late erythroid progenitors [35]. How the globin chain imbalance might affect the rate of heme synthesis is still a matter of investigation. The reduction of heme biosynthesis in *β*-thalassemic erythropoiesis has nevertheless a positive action to prevent the cytotoxic effect of free heme excess. Other cytoprotective mechanisms in response to oxidative stress in *β*-thalassemic erythroid cells also probably involve PRDX2 protein. PRDX2 is abundantly expressed during *β*-thalassemic erythropoiesis and binds heme in erythroid precursors, possibly playing an additional role to protect maturing cells by free heme from apoptosis [35].

Another protective factor in *β*-thalassemic erythropoiesis involves the heme-regulated inhibitor of protein translation, which represses globin translation in heme-deficient erythroid precursors. Heme-regulated inhibitor of protein translation plays a role in murine *β*-thalassemia, since anemia is more severe in *β*-thalassemic mice genetically lacking this protein [63, 64]. Roles of heme or heme inhibitors in ineffective erythropoiesis are still not known.

### 4.6. Role of Inflammatory Cytokines in Ineffective Erythropoiesis in *β*-Thalassemia Patients

Increased level of several inflammatory cytokines has been reported in *β*-TM and might contribute to ineffective erythropoiesis, through the well-known mechanism of “anemia associated with a chronic disease.”

Further studies have shown an increased of TNF-*α* concentration in *β*-TM patients, unrelated to splenectomy [65, 66] or only in the splenectomised patients group [67–69]. In these studies, the authors suggested that the main cause for TNF-*α* rise was macrophage activation due to iron overload and the antigenic stimulation induced by chronic transfusion therapy. 

TNF-*α* inhibits erythropoiesis *in vivo* and *in vitro* [70–80]. However, the mechanism by which TNF-*α* inhibits erythroid progenitor cells remains unclear. TNF-*α* induces an increase of apoptosis within the compartment of immature erythroblasts and a decrease in mature erythroblasts. TNF-*α* inhibits directly the BFU-E colony growth [70, 79, 80] whereas inhibition of CFU-E colony growth by the TNF-*α* is indirect via stimulation of *β*-interferon production from accessory cells [81, 82]. TNF-*α* might act directly on the TNF receptor expressed on immature erythroblasts or by inducing ceramide synthesis, lipids component of the cell membrane which can act as a signaling molecule involved in TNF-induced apoptosis. It was also reported that the inhibitory effect of TNF-*α* on erythroid maturation might be involved in NF-*κ*B induction [70]. TNF-*α* cannot only directly inhibit erythroid differentiation but also facilitate proliferation of nonerythroid precursor cells (such as dendritics cells) in chronic disease with inflammatory syndrome [80]. 

 Furthermore, it was described that transforming growth factor-*β* (TGF-*β*) plasma level was higher in *β*-TM splenectomized patients as compared to control group as well as other cytokines from TGF-*β* superfamily [83]. Therefore, TGF-*β* is a paradoxical inhibitor of normal erythropoiesis that acts by blocking proliferation and accelerating differentiation of erythroid progenitors [84]. Its potential role in pathophysiological mechanisms of *β*-thalassemia ineffective erythropoiesis has not been studied to date. 

### 4.7. Other Pathophysiological Pathways Involved in *β*-Thalassemia Ineffective Erythropoiesis

#### 4.7.1. Macrophages Number and Activation Are Enhanced

As described above, macrophages number and level of activation are enhanced in bone marrow of *β*-thalassemic patients [29, 37]. It was suggested that macrophages activation was due to iron overload and antigenic stimulation related to chronic transfusion therapy [83]. Those activated macrophages selectively phagocyte apoptotic erythroblasts exhibiting “eat me signal” [37, 38, 85], thereby contributing to the ineffective erythropoiesis. 

It has been demonstrated that thalassemic erythrocytes are phagocytosed by activated macrophages *in vitro* [85], and the mean number of *β*-thalassemic cells ingested by monocytes was found to be approximatively 30% higher than that for normal monocytes [86]. Mechanisms involved in the recognition of apoptotic erythroblasts by macrophages are not fully understood, but CD36 at the surface of macrophages and phosphatidyl serine residues exposed on apoptotic erythroblasts membrane appear to be involved [37]. Furthermore, oxidant injury that produced a IgG-bound neoantigen band 3, associated to complement, provided signals for macrophages to remove such affected erythroblasts [53].

#### 4.7.2. Epo Response to Anemia in *β*-Thalassemia Major

In *β*-thalassemia, Epo is dramatically increased in response to anemia and hypoxia [87]. Nevertheless, Epo response is blunted as compared with Epo response in aplastic anemia or iron deficiency anemia [88, 89]. Underlying mechanisms for the blunted Epo response in patients with *β*-TM are not well understood. Three hypotheses are suggested:

- increased capture and faster clearance of Epo by erythroid cells hyperplasia [90],
- inhibition of Epo renal synthesis by inflammatory cytokines (IL-1*α*, IL-1*β*, TGF-*β*, or TNF-*α*) [91] as showed in other anemias of chronic disease [92–96] or by impaired renal function [89, 97–101]; very high serum Epo levels were found in young patients with *β*-TM and *β*-thalassemia intermedia, comparable to the levels found in patients with aplastic anemia, which were different from the levels found in older thalassemia patients with the same degree of anemia; it is suggested, therefore, that a decrease in serum Epo levels could develop during the course of the disease [102, 103],
- The low oxygen-Hb affinity and subsequent right shifting of oxygen-Hb dissociation curve would facilitate tissue oxygen availability, decreasing the hypoxic burden to anemia. Both low oxygen-Hb affinity and increased 2,3-diphosphoglycerate levels [104] present in *β*-TM might induce an inadequate Epo response to a given degree of anemia.

Nevertheless, increased Epo level, secondary to profound anemia, is believed to be the cause of early erythroid progenitors and precursors expansion in *β*-TM [87]. In *β*-TM mice model, it was shown that persistent activation of JAK2, as a consequence of high levels of Epo, drives erythroid expansion and extramedullary hematopoiesis, thus might constitute a target, by using JAK2 inhibitors, to treat this complication and decrease transfusion burden [105].

#### 4.7.3. Iron Overload in Thalassemia

Tissue iron overload is the most important complication of *β*-thalassemia and is a major focus of therapeutic management [42, 106]. 

Blood transfusion is a comprehensive source of iron loading for *β*-thalassemia patients. Nevertheless, iron overload occurs also in patients who have not received transfusions such as patients suffering from thalassemia intermedia [42, 107, 108]. Decreased levels of hepcidin in these patients explain this paradoxical feature. Hepcidin is a key regulator of iron homeostasis: it blocks iron release from macrophages and hepatocytes and inhibits intestinal iron absorption. Its liver expression increases in response to iron overload and inflammatory stimuli [4, 109]. If hepcidin expression would be correctly regulated, it should be increased in *β*-thalassemia patients in order to decrease intestinal iron absorption. However, the opposite effect is observed [110, 111]. Indeed, two hepcidin erythroid regulators have been reported: the growth differentiation factor 15 (GDF15) and the twisted gastrulation protein homolog 1 (TWSG1) [112, 113]. High concentrations of both proteins, members of the TGF-*β* superfamily, were evidenced in *β*-thalassemia serum compared to normal human serum. These proteins downregulate hepcidin secretion by hepatocytes [112, 114]. GDF15 expression is associated with cellular stress and apoptosis and is expressed at low level during normal erythropoiesis. While *β*-thalassemic erythroid differentiation, GDF15 has been shown to be secreted by apoptotic erythroid cells at final stages. In contrast, the highest levels of TWSG1 were detected at early stages of erythroblast differentiation, before hemoglobinization [113]. 

Red blood cell membranes from thalassemic patients carry abnormal deposits of iron, presumed to mediate a variety of oxidative induced membrane dysfunctions. The combination of iron overload and increased outpouring of catabolic iron from the reticuloendothelial system overwhelms the iron-carrying reticuloendothelial system and the iron capacity of transferrin, the main transport protein, to bind and detoxify iron. Non-transferrin-bound fraction of plasma iron might promote the generation of malonyldialdehyde [115] and free hydroxyl radicals, propagators of oxygen-related damage [42, 116, 117]. In addition, several pathobiochemical consequences in thalassemic RBC membranes, such as increased lipid peroxidation and protein thiol oxidation, have been linked to the deposition of generic iron on the cytosolic leaflet of plasma membrane. This mechanism could also contribute to erythroblast apoptosis [118]. 

It has been shown in mouse model of *β*-thalassemia intermedia that decreasing iron availability of erythroid cells limits the formation of toxic alpha-chain/heme aggregates and improves ineffective erythropoiesis and anemia [119].

Moreover, it was hypothesized that oral iron chelators, which have an enhanced capacity to penetrate through cell membrane, might be useful in chelating these pathologic iron deposits responsible for ROS generation [118]. This suggestion receives further support from *in vitro* and some *in vivo* studies using these treatments. It was shown that membrane free-iron content decreased as did heme content of RBC membranes from deferiprone-treated thalassemic patients [118]. It has been recently described that deferasirox therapy in *β*-TM patients is associated with higher levels of circulating erythroid burst-forming unit than controls and other iron chelators [120].

Furthermore, *β*-TM patients with severe hemochromatosis may develop severe endocrine complications due to iron overload. Hypogonadism, hypothyroidism, and hypoadrenalism may also contribute to anemia. Thus endocrinopathy must be monitored regularly and treated with hormone replacement [121]. 

#### 4.7.4. Masked Deficit of Folic Acid in Thalassemia

Folic acid deficiency has been reported in both thalassemia major and minor [122–124], as a consequence of increased folate use caused by increased erythropoiesis. It can lead to overestimation of RBC deficiency. Daily folate supplementation is currently advised for patients with hemoglobinopathy [124]. 

## 5. Conclusion

The pathophysiological mechanisms of ineffective erythropoiesis in *β*-thalassemia could be the conjunction of several mechanisms of which the final consequence is the arrest of maturation and increased apoptosis of erythroblasts during their terminal differentiation stage. 

Putative actors of ineffective erythropoiesis are suggested to be (1) oxidative stress induced by the excess of *α*-globin secondary to the *α*/*β* globin imbalance, (2) iron overload, and (3) endocrines and cytokine and environmental factors. Key questions still remain to be addressed: how deposition of *α*-globin chains and/or ROS production in erythroid precursors induce apoptosis and if antiapoptotic processes (involving heat shock protein) in erythroid cells are deficient or overwhelm. In the future it will be critical to decipher the precise role and mechanisms of these components in order to understand the ineffective erythropoiesis in *β*-thalassemia and to develop new therapeutic strategies based on these potential targets.

## References

[1] Weatherall DJ (2001). Phenotype-genotype relationships in monogenic disease: lessons from the thalassaemias. *Nature Reviews Genetics*.

[2] Khandros E, Thom CS, D'Souza J, Weiss MJ (2012). Integrated protein quality-control pathways regulate free *α*-globin in murine *β*-thalassemia. *Blood*.

[3] Khandros E, Weiss MJ (2010). Protein quality control during erythropoiesis and hemoglobin synthesis. *Hematology/Oncology Clinics of North America*.

[4] Rund D, Rachmilewitz E (2005). *β*-thalassemia. *The New England Journal of Medicine*.

[5] Schrier SL (2002). Pathophysiology of thalassemia. *Current Opinion in Hematology*.

[6] Gill FM, Schwartz E (1973). Free *α*-globin pool in human bone marrow. *Journal of Clinical Investigation*.

[7] Shaeffer JR (1967). Evidence for soluble a chains as intermediates in hemoglobin synthesis in the rabbit reticulocyte. *Biochemical and Biophysical Research Communications*.

[8] Gregory T, Yu C, Ma A, Orkin SH, Blobel GA, Weiss MJ (1999). GATA-1 and erythropoietin cooperate to promote erythroid cell survival by regulating bcl-x(L) expression. *Blood*.

[9] Zermati Y, Garrido C, Amsellem S (2001). Caspase activation is required for terminal erythroid differentiation. *Journal of Experimental Medicine*.

[10] Ribeil JA, Zermati Y, Vandekerckhove J (2007). Hsp70 regulates erythropoiesis by preventing caspase-3-mediated cleavage of GATA-1. *Nature*.

[11] Allen TD, Dexter TM (1982). Ultrastructural aspects of erythropoietic differentiation in long-term bone marrow culture. *Differentiation*.

[12] Chasis JA, Mohandas N (2008). Erythroblastic islands: niches for erythropoiesis. *Blood*.

[13] Fang J, Menon M, Kapelle W (2007). EPO modulation of cell-cycle regulatory genes, and cell division, in primary bone marrow erythroblasts. *Blood*.

[14] Socolovsky M, Murrell M, Liu Y, Pop R, Porpiglia E, Levchenko A (2007). Negative autoregulation by FAS mediates robust fetal erythropoiesis. *PLoS Biology*.

[15] Menon MP, Karur V, Bogacheva O, Bogachev O, Cuetara B, Wojchowski DM (2006). Signals for stress erythropoiesis are integrated via an erythropoietin receptor-phosphotyrosine-343-Stat5 axis. *Journal of Clinical Investigation*.

[16] Hengartner MO (2000). The biochemistry of apoptosis. *Nature*.

[17] Yi CH, Yuan J (2009). The jekyll and hyde functions of caspases. *Developmental Cell*.

[18] Locksley RM, Killeen N, Lenardo MJ (2001). The TNF and TNF receptor superfamilies: Integrating mammalian biology. *Cell*.

[19] Orrenius S (2004). Mitochondrial regulation of apoptotic cell death. *Toxicology Letters*.

[20] Green DR, Kroemer G (2004). The pathophysiology of mitochondrial cell death. *Science*.

[21] de Maria R, Zeuner A, Eramo A (1999). Negative regulation of erythropoiesis by caspase-mediated cleavage of GATA-1. *Nature*.

[22] Zeuner A, Eramo A, Testa U (2003). Control of erythroid cell production via caspase-mediated cleavage of transcription factor SCL/Tal-1. *Cell Death and Differentiation*.

[23] de Maria R, Testa U, Luchetti L (1999). Apoptotic role of Fas/Fas ligand system in the regulation of erythropoiesis. *Blood*.

[24] Koulnis M, Liu Y, Hallstrom K, Socolovsky M (2011). Negative autoregulation by fas stabilizes adult erythropoiesis and accelerates its stress response. *PLoS ONE*.

[25] Testa U (2004). Apoptotic mechanisms in the control of erythropoiesis. *Leukemia*.

[26] Finch CA, Sturgeon P (1957). Erythrokinetics in Cooley’s anemia. *Blood*.

[27] Pootrakul P, Sirankapracha P, Hemsorach S (2000). A correlation of erythrokinetics, ineffective erythropoiesis, and erythroid precursor apoptosis in Thai patients with thalassemia. *Blood*.

[28] Finch CA, Deubelbeiss K, Cook JD (1970). Ferrokinetics in man. *Medicine*.

[29] Centis F, Tabellini L, Lucarelli G (2000). The importance of erythroid expansion in determining the extent of apoptosis in erythroid precursors in patients with *β*-thalassemia major. *Blood*.

[30] Yuan J, Angelucci E, Lucarelli G (1993). Accelerated programmed cell death (apoptosis) in erythroid precursors of patients with severe *β*-thalassemia (Cooley’s anemia). *Blood*.

[31] Schrier SL (1997). Pathophysiology of the thalassemias the Albion Walter Hewlett Award presentation. *Western Journal of Medicine*.

[32] Mathias LA, Fisher TC, Zeng L (2000). Ineffective erythropoiesis in *β*-thalassemia major is due to apoptosis at the polychromatophilic normoblast stage. *Experimental Hematology*.

[33] Wickramasinghe SN, Bush V (1975). Observations on the ultrastructure of erythropoietic cells and reticulum cells in the bone marrow of patients with homozygous *β* thalassaemia. *The British Journal of Haematology*.

[34] Leecharoenkiat A, Wannatung T, Lithanatudom P (2011). Increased oxidative metabolism is associated with erythroid precursor expansion in *β*0-thalassaemia/Hb E disease. *Blood Cells, Molecules and Diseases*.

[35] de Franceschi L, Bertoldi M, de Falco L (2011). Oxidative stress modulates heme synthesis and induces peroxiredoxin-2 as a novel cytoprotective response in *β*-thalassemic erythropoiesis. *Haematologica*.

[36] Lacombe C, Da Silva JL, Bruneval P (1988). Peritubular cells are the site of erythropoietin synthesis in the murine hypoxic kidney. *Journal of Clinical Investigation*.

[37] Angelucci E, Bai H, Centis F (2002). Enhanced macrophagic attack on *β*-thalassemia major erythroid precursors. *Haematologica*.

[38] Kuypers FA, de Jong K (2004). The role of phosphatidylserine in recognition and removal of erythrocytes. *Cellular and Molecular Biology*.

[39] Liu Y, Pop R, Sadegh C, Brugnara C, Haase VH, Socolovsky M (2006). Suppression of Fas-FasL coexpression by erythropoietin mediates erythroblast expansion during the erythropoietic stress response in vivo. *Blood*.

[40] Schrier SL, Centis F, Verneris M, Ma L, Angelucci E (2003). The role of oxidant injury in the pathophysiology of human thalassemias. *Redox Report*.

[41] Nathan DG, Gunn RB (1966). Thalassemia: the consequences of unbalanced hemoglobin synthesis. *The American Journal of Medicine*.

[42] Olivieri NF (1999). The *β*-thalassemias. *The New England Journal of Medicine*.

[43] Tavazzi D, Duca L, Graziadei G, Comino A, Fiorelli G, Cappellini MD (2001). Membrane-bound iron contributes to oxidative damage of *β*-thalassaemia intermedia erythrocytes. *The British Journal of Haematology*.

[44] Bank A (1968). Hemoglobin synthesis in *β*-thalassemia: the properties of the free alpha-chains. *Journal of Clinical Investigation*.

[45] Kong Y, Zhou S, Kihm AJ (2004). Loss of *α*-hemoglobin-stabilizing protein impairs erythropoiesis and exacerbates *β*-thalassemia. *Journal of Clinical Investigation*.

[46] Shinar E, Rachmilewitz EA (1990). Oxidative denaturation of red blood cells in thalassemia. *Seminars in Hematology*.

[47] Fujisawa T, Takeda K, Ichijo H (2007). ASK family proteins in stress response and disease. *Molecular Biotechnology*.

[48] Circu ML, Aw TY (2010). Reactive oxygen species, cellular redox systems, and apoptosis. *Free Radical Biology and Medicine*.

[49] Nagata M, Arimitsu N, Ito T, Sekimizu K (2007). Antioxidant N-acetyl-l-cysteine inhibits erythropoietin-induced differentiation of erythroid progenitors derived from mouse fetal liver. *Cell Biology International*.

[50] Schrier SL, Rachmilewitz E, Mohandas N (1989). Cellular and membrane properties of *α* and *β* thalassemic erythrocytes are different: implication for differences in clinical manifestations. *Blood*.

[51] Advani R, Sorenson S, Shinar E, Lande W, Rachmilewitz E, Schrier SL (1992). Characterization and comparison of the red blood cell membrane damage in severe human *α*- and *β*-thalassemia. *Blood*.

[52] Aljurf M, Ma L, Angelucci E (1996). Abnormal assembly of membrane proteins in erythroid progenitors of patients with *β*-thalassemia major. *Blood*.

[53] Yuan J, Kannan R, Shinar E, Rachmilewitz EA, Low PS (1992). Isolation, characterization, and immunoprecipitation studies of immune complexes from membranes of *β*-thalassemic erythrocytes. *Blood*.

[54] Kihm AJ, Kong Y, Hong W (2002). An abundant erythroid protein that stabilizes free *α*-haemoglobin. *Nature*.

[55] Dos Santos CO, Duarte ASS, Saad STO, Costa FF (2004). Expression of *α*-hemoglobin stabilizing protein gene during human erythropoiesis. *Experimental Hematology*.

[56] Dos Santos CO, Dore LC, Valentine E (2008). An iron responsive element-like stem-loop regulates *α*-hemoglobin-stabilizing protein mRNA. *The Journal of Biological Chemistry*.

[57] Weiss MJ, Dos Santos CO (2009). Chaperoning erythropoiesis. *Blood*.

[58] Yu X, Kong Y, Dore LC (2007). An erythroid chaperone that facilitates folding of *α*-globin subunits for hemoglobin synthesis. *Journal of Clinical Investigation*.

[59] Feng L, Zhou S, Gu L (2005). Structure of oxidized *α*-haemoglobin bound to AHSP reveals a protective mechanism for haem. *Nature*.

[60] Zhou S, Olson JS, Fabian M, Weiss MJ, Gow AJ (2006). Biochemical fates of *α* hemoglobin bound to *α* hemoglobin-stabilizing protein AHSP. *The Journal of Biological Chemistry*.

[61] Dos Santos CO, Zhou S, Secolin R (2008). Population analysis of the alpha hemoglobin stabilizing protein (AHSP) gene identifies sequence variants that alter expression and function. *American Journal of Hematology*.

[62] Viprakasit V, Tanphaichitr VS, Chinchang W, Sangkla P, Weiss MJ, Higgs DR (2004). Evaluation of *α* hemoglobin stabilizing protein (AHSP) as a genetic modifier in patients with *β* thalassemia. *Blood*.

[63] Han AP, Fleming MD, Chen JJ (2005). Heme-regulated eIF2*α* kinase modifies the phenotypic severity of murine models of erythropoietic protoporphyria and *β*-thalassemia. *Journal of Clinical Investigation*.

[64] Chen JJ (2007). Regulation of protein synthesis by the heme-regulated eIF2*α* kinase: relevance to anemias. *Blood*.

[65] Lombardi G, Matera R, Minervini MM (1994). Serum levels of cytokines and soluble antigens in polytransfused patients with *β*-thalassemia major: relationship to immune status. *Haematologica*.

[66] Meliconi R, Uguccioni M, Lalli E (1992). Increased serum concentrations of tumour necrosis factor in *β* thalassaemia: effect of bone marrow transplantation. *Journal of Clinical Pathology*.

[67] Chuncharunee S, Archararit N, Hathirat P, Udomsubpayakul U, Atichartakarn V (1997). Levels of serum interleukin-6 and tumor necrosis factor in postsplenectomized thalassemic patients. *Journal of the Medical Association of Thailand*.

[68] Gharagozloo M, Karimi M, Amirghofran Z (2009). Double-faced cell-mediated immunity in *β*-thalassemia major: stimulated phenotype versus suppressed activity. *Annals of Hematology*.

[69] Del Vecchio GC, Schettini F, Piacente L, De Santis A, Giordano P, de Mattia D (2002). Effects of deferiprone on immune status and cytokine pattern in thalassaemia major. *Acta Haematologica*.

[70] Xiao W, Koizumi K, Nishio M (2002). Tumor necrosis factor-*α* inhibits generation of glycophorin A^+^ cells by CD34^+^ cells. *Experimental Hematology*.

[71] Roodman GD, Bird A, Hutzler D, Montgomery W (1987). Tumor necrosis factor-alpha and hematopoietic progenitors: effects of tumor necrosis factor on the growth of erythroid progenitors CFU-E and BFU-E and the hematopoietic cell lines K562, HL60, and HEL cells. *Experimental Hematology*.

[72] Johnson RA, Waddelow TA, Caro J, Oliff A, Roodman GD (1989). Chronic exposure to tumor necrosis factor in vivo preferentially inhibits erythropoiesis in nude mice. *Blood*.

[73] Broxmeyer HE, Williams DE, Lu L (1986). The suppressive influences of human tumor necrosis factors on bone marrow hematopoietic progenitor cells from normal donors and patients with leukemia: synergism of tumor necrosis factor and interferon-*γ*. *Journal of Immunology*.

[74] Murase T, Hotta T, Saito H, Ohno R (1987). Effect of recombinant human tumor necrosis factor on the colony growth of human leukemia progenitor cells and normal hematopoietic progenitor cells. *Blood*.

[75] Backx B, Broeders L, Bot FJ, Löwenberg B (1991). Positive and negative effects of tumor necrosis factor on colony growth from highly purified normal marrow progenitors. *Leukemia*.

[76] Rusten LS, Jacobsen SEW (1995). Tumor necrosis factor (TNF)-*α* directly inhibits human erythropoiesis in vitro: role of p55 and p75 TNF receptors. *Blood*.

[77] Blick M, Sherwin SA, Rosenblum M, Gutterman J (1987). Phase I study of recombinant tumor necrosis factor in cancer patients. *Cancer Research*.

[78] Papadaki HA, Kritikos HD, Valatas V, Boumpas DT, Eliopoulos GD (2002). Anemia of chronic disease in rheumatoid arthritis is associated with increased apoptosis of bone marrow erythroid cells: improvement following anti-tumor necrosis factor-*α* antibody therapy. *Blood*.

[79] Li XF, Anderson J, Hutzler D, Roodman GD (1989). Hemin-induced erythroid differentiation changes the sensitivity of K562 cells to tumor necrosis factor-*α*. *Experimental Hematology*.

[80] Fukaya H, Xiao W, Inaba K (2004). Codevelopment of dendritic cells along with erythroid differentiation from human CD34^+^ cells by tumor necrosis factor-*α*. *Experimental Hematology*.

[81] Means RT, Dessypris EN, Krantz SB (1990). Inhibition of human colony-forming-unit erythroid by tumor necrosis factor requires accessory cells. *Journal of Clinical Investigation*.

[82] Means RT, Krantz SB (1993). Inhibition of human erythroid colony-forming units by tumor necrosis factor requires *β* interferon. *Journal of Clinical Investigation*.

[83] Moshtaghi-Kashanian GR, Gholamhoseinian A, Hoseinimoghadam A, Rajabalian S (2006). Splenectomy changes the pattern of cytokine production in *β*-thalassemic patients. *Cytokine*.

[84] Zermati Y, Varet B, Hermine O (2000). TGF-*β*1 drives and accelerates erythroid differentiation in the epo-dependent UT-7 cell line even in the absence of erythropoietin. *Experimental Hematology*.

[85] Knyszynski A, Danon D, Kahane I, Rachmilewitz EA (1979). Phagocytosis of nucleated and mature *β* thalassaemic red blood cells by mouse macrophages in vitro. *The British Journal of Haematology*.

[86] Wanachiwanawin W, Siripanyphinyo U, Fucharoen S (1993). Activation of monocytes for the immune clearance of red cells in *β*(o)-thalassaemia/HbE. *The British Journal of Haematology*.

[87] Ginzburg Y, Rivella S (2011). *β*-thalassemia: a model for elucidating the dynamic regulation of ineffective erythropoiesis and iron metabolism. *Blood*.

[88] Cazzola M, Guarnone R, Cerani P, Centenara E, Rovati A, Beguin Y (1998). Red blood cell precursor mass as an independent determinant of serum erythropoietin level. *Blood*.

[89] Chen JS, Lin KH, Wang ST, Tsao CJ, Yeh TF (1998). Blunted serum erythropoietin response to anemia in patients polytransfused for *β*-thalassemia major. *Journal of Pediatric Hematology/Oncology*.

[90] Camaschella C, Gonetta S, Calabrese R (1996). Serum erythropoietin and circulating transferrin receptor in thalassemia intermedia patients with heterogeneous genotypes. *Haematologica*.

[91] Faquin WC, Schneider TJ, Goldberg MA (1992). Effect of inflammatory cytokines on hypoxia-induced erythropoietin production. *Blood*.

[92] Hochberg MC, Arnold CM, Hogans BB, Spivak JL (1988). Serum immunoreactive erythropoietin in rheumatoid arthritis: impaired response to anemia. *Arthritis and Rheumatism*.

[93] Miller CB, Jones RJ, Piantadosi S, Abeloff MD, Spivak JL (1990). Decreased erythropoietin response in patients with the anemia of cancer. *The New England Journal of Medicine*.

[94] Baer AN, Dessypris EN, Goldwasser E, Krantz SB (1987). Blunted erythropoietin response to anaemia in rheumatoid arthritis. *The British Journal of Haematology*.

[95] Johannsen H, Jelkmann W, Wiedemann G, Otte M, Wagner T (1989). Erythropoietin/haemoglobin relationship in leukaemia and ulcerative colitis. *European Journal of Haematology*.

[96] Spivak JL, Barnes DC, Fuchs E, Quinn TC (1989). Serum immunoreactive erythropoietin in HIV-infected patients. *The Journal of the American Medical Association*.

[97] Sumboonnanonda A, Malasit P, Tanphaichitr VS (1998). Renal tubular function in *β*-thalassemia. *Pediatric Nephrology*.

[98] Aldudak B, Bayazit AK, Noyan A (2000). Renal function in pediatric patients with *β*-thalassemia major. *Pediatric Nephrology*.

[99] Cianciulli P, Sollecito D, Sorrentino F (1994). Early detection of nephrotoxic effects in thalassemic patients receiving desferrioxamine therapy. *Kidney International*.

[100] Landing BH, Gonick HC, Nadorra RL (1989). Renal lesions and clinical findings in thalassemia major and other chronic anemias with hemosiderosis. *Pediatric Pathology*.

[101] Smolkin V, Halevy R, Levin C (2008). Renal function in children with *β*-thalassemia major and thalassemia intermedia. *Pediatric Nephrology*.

[102] Manor D, Fibach E, Goldfarb A, Rachmilewitz EA (1986). Erythropoietin activity in the serum of *β* thalassemic patients. *Scandinavian Journal of Haematology*.

[103] Rachmilewitz EA, Aker M (1998). The role of recombinant human erythropoietin in the treatment of thalassemia. *Annals of the New York Academy of Sciences*.

[104] Ricci G, Castaldi G, Zavagli G, Lupi G, Turati A, Bezzi T (1982). Red cell 2,3-diphosphoglycerate contents and oxygen affinity in heterozygous *β*-thalassaemia. *Acta Haematologica*.

[105] Libani IV, Guy EC, Melchiori L (2008). Decreased differentiation of erythroid cells exacerbates ineffective erythropoiesis in *β*-thalassemia. *Blood*.

[106] Olivieri NF, Brittenham GM (1997). Iron-chelating therapy and the treatment of thalassemia. *Blood*.

[107] Pippard MJ, Callender ST, Warner GT, Weatherall DJ (1979). Iron absorption and loading in *β*-thalassaemia intermedia. *The Lancet*.

[108] Pootrakul P, Kitcharoen K, Yansukon P (1988). The effect of erythroid hyperplasia on iron balance. *Blood*.

[109] Ganz T (2003). Hepcidin, a key regulator of iron metabolism and mediator of anemia of inflammation. *Blood*.

[110] Papanikolaou G, Tzilianos M, Christakis JI (2005). Hepcidin in iron overload disorders. *Blood*.

[111] Kattamis A, Papassotiriou I, Palaiologou D (2006). The effects of erythropoetic activity and iron burden on hepcidin expression in patients with thalassemia major. *Haematologica*.

[112] Tanno T, Bhanu NV, Oneal PA (2007). High levels of GDF15 in thalassemia suppress expression of the iron regulatory protein hepcidin. *Nature Medicine*.

[113] Tanno T, Porayette P, Sripichai O (2009). Identification of TWSG1 as a second novel erythroid regulator of hepcidin expression in murine and human cells. *Blood*.

[114] Kanda J, Mizumoto C, Kawabata H (2008). Serum hepcidin level and erythropoietic activity after hematopoietic stem cell transplantation. *Haematologica*.

[115] Cighetti G, Duca L, Bortone L (2002). Oxidative status and malondialdehyde in *β*-thalassaemia patients. *European Journal of Clinical Investigation*.

[116] Hershko C, Weatherall DJ (1988). Iron-chelating therapy. *Critical Reviews in Clinical Laboratory Sciences*.

[117] Hershko C, Konijn AM, Link G (1998). Iron chelators for thalassaemia. *The British Journal of Haematology*.

[118] Shalev O, Repka T, Goldfarb A (1995). Deferiprone (L1) chelates pathologic iron deposits from membranes of intact thalassemic and sickle red blood cells both in vitro and in vivo. *Blood*.

[119] Li H, Rybicki AC, Suzuka SM (2010). Transferrin therapy ameliorates disease in beta-thalassemic mice. *Nature Medicine*.

[120] Forni GL, Podesta' M, Musso M (2012). Differential effects of the type of iron chelator on the absolute number of hematopoietic peripheral progenitors in patients with *β*-thalassemia major. *Haematologica*.

[121] Tiosano D, Hochberg Z (2001). Endocrine complications of thalassemia. *Journal of Endocrinological Investigation*.

[122] Danel P, Girot R, Tchernia G (1983). Thalassemia major presenting as megaloblastic anemia with folate deficiency. *Archives Francaises de Pediatrie*.

[123] Ortuño F, Remacha A, Martin S, Soler J, Gimferrer E (1990). Prevalence of folate deficiency in *β* and delta-beta heterozygous thalassemia. *Haematologica*.

[124] Mazzone A, Vezzoli M, Ottini E (2001). Masked deficit of B12 and folic acid in thalassemia. *American Journal of Hematology*.

